# Non-classical B Cell Memory of Allergic IgE Responses

**DOI:** 10.3389/fimmu.2019.00715

**Published:** 2019-04-26

**Authors:** Sean P. Saunders, Erica G. M. Ma, Carlos J. Aranda, Maria A. Curotto de Lafaille

**Affiliations:** ^1^Division of Pulmonary, Critical Care and Sleep Medicine, Laboratory of Allergy and Inflammation, Department of Medicine, New York University, New York, NY, United States; ^2^Sackler Institute of Graduate Biomedical Sciences, New York University, New York, NY, United States; ^3^Department of Cell Biology, New York University School of Medicine, New York, NY, United States

**Keywords:** allergy, IgE, memory B cells, plasma cells, anaphylaxis, sequential switching, IgG

## Abstract

The long-term effectiveness of antibody responses relies on the development of humoral immune memory. Humoral immunity is maintained by long-lived plasma cells that secrete antigen-specific antibodies, and memory B cells that rapidly respond to antigen re-exposure by generating new plasma cells and memory B cells. Developing effective immunological memory is essential for protection against pathogens, and is the basis of successful vaccinations. IgE responses have evolved for protection against helminth parasites infections and against toxins, but IgE is also a potent mediator of allergic diseases. There has been a dramatic increase in the incidence of allergic diseases in recent decades and this has provided the impetus to study the nature of IgE antibody responses. As will be discussed in depth in this review, the IgE memory response has unique features that distinguish it from classical B cell memory.

## General Mechanisms of Humoral Memory in Mice and Humans

IgE antibodies mediate allergic diseases through their ability to bind to high-affinity receptors on mast cells and induce degranulation upon allergen crosslinking (1). Given the increasing prevalence of allergic reactions and allergic diseases, the study of human IgE cells becomes extremely important. Furthermore, the beneficial effect of anti-IgE treatment on allergic asthma and other chronic allergic diseases validated IgE as a therapeutic target (2). While a high titer of serum IgE has long been considered the cardinal marker of atopy, IgE-producing cells are extremely rare in humans and mice, suggesting that IgE production is strongly regulated. IgE antibodies also have protective functions against parasite infections and toxins (3, 4). Possibly as a consequence of this dual beneficial and pathogenic potential, IgE production has been evolutionary conserved but is strongly regulated. Despite the importance of IgE in allergic pathology, very little is known about the origin of human IgE B cells and the mechanisms of humoral IgE memory.

**Figure 1:**
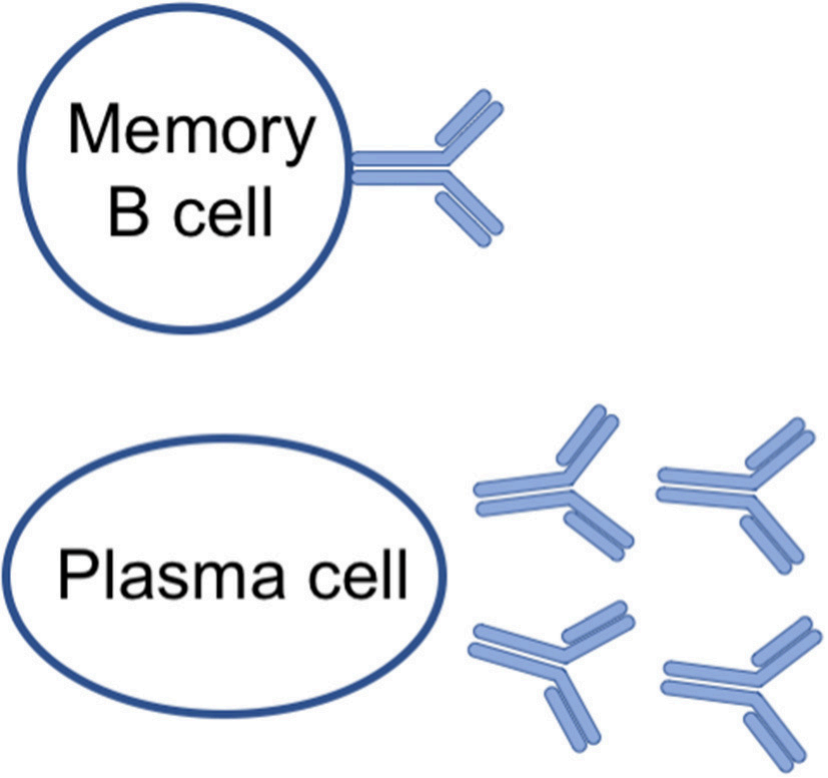
Cellular components of B cell memory.

The classical humoral memory of IgM, IgGs and IgA antibodies is mediated by a two-pronged mechanism (5): non-dividing quiescent memory B cells that can be quickly reactivated, and long-lived plasma cells (6, 7) that constantly secrete antibodies during their lifespan (Figure 1 and Table 1).

**Table 1:** Memory B cells and long-lived plasma cells are the cellular components of B cell memory.

**Characteristic**	**Memory B cells**	**Long-lived plasma cells**
Cell division	Non-dividing	Non-dividing
Activation	Respond with proliferation and differentiation	Do not respond to activation
Differentiation potential	Germinal center cells Plasma cells Memory cells	Terminally differentiated into antibody secretory cells
Tissue location	Circulating between blood and lymphoid organs	Bone marrow and other specialized niches

*Comparison of their main characteristics*.

Affinity maturation and class switch recombination of antibody genes are the central processes in the establishment of effective B cell memory. Affinity maturation is the mechanism by which B cells improve the recognition of their cognate antigen by refining the affinity of their B cell receptor (BCR). This process occurs in germinal centers and involves the somatic mutation of V(D)J genes followed by selection of higher affinity B cell clones through interaction with both antigen-loaded follicular dendritic cells (FDC) and T follicular helper cells (Tfh) (8–11). Both plasma cells of higher affinity (12), and memory B cells of broad affinity range (13–16) emerge from the germinal center reaction.

**Figure 2:**
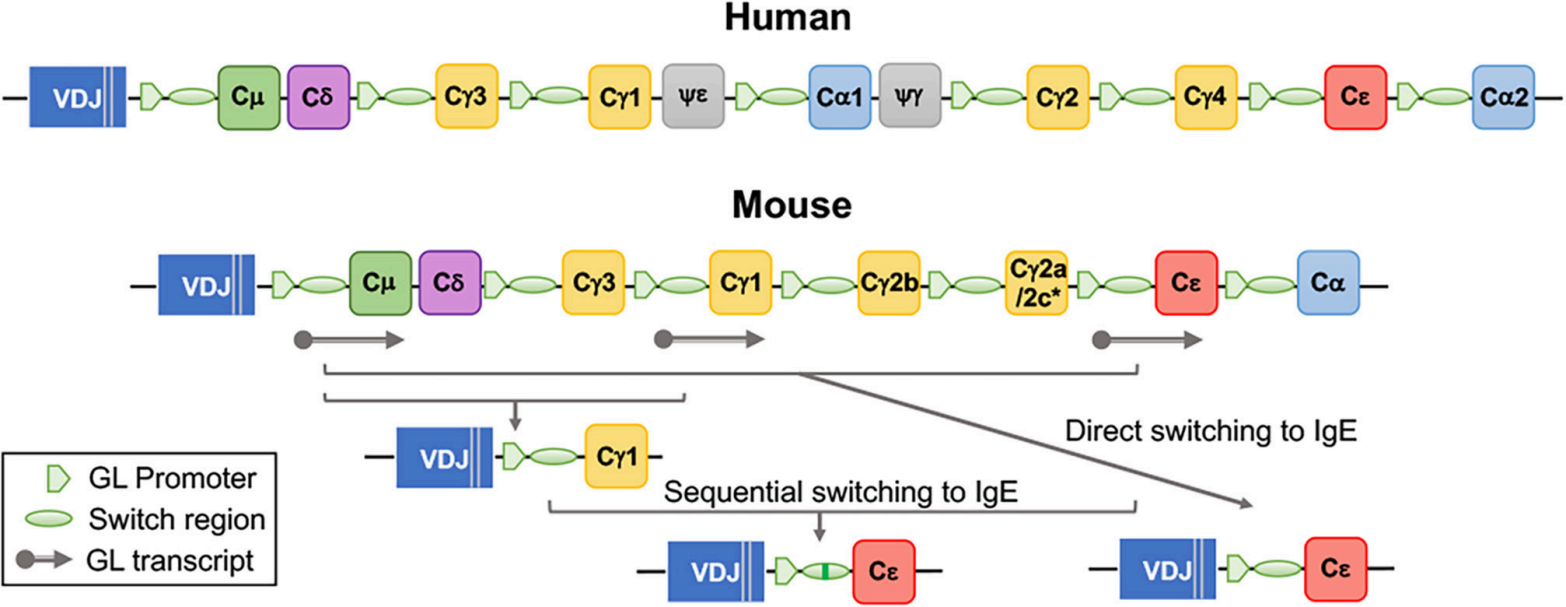
Organization of immunoglobulin heavy chain constant region genes in human and mouse and generation of IgE by direct and sequential class switching. The gene encoding the constant region (C) of IgE, Cε, is localized at the 3' end of the locus, upstream of Cα2 in humans and of Cα in mice. ^*^Mice of the BALB/c strain carry a Cγ2a gene, while mice of the C57/Bl strains carry the Cγ2c gene. Class switch recombination to IgE is initiated by pSTAT6 dependent production of the GL (germline) Cε transcript. Direct class switching occurs by recombination between Sμ and Sε regions. Sequential switching involves a two-step recombination, an initial recombination to IgG1, and a second recombination to IgE.

Immunoglobulin class switch recombination (9, 17) is the mechanism whereby activated B cells “switch” their constant region while maintaining their antigen-binding domain, resulting in the production of IgG, IgA, and IgE antibodies (Figure 2). While V(D)J domains determine antigen specificity, the constant regions endow the antibodies with specific biological activities (18). In mice there are four IgG isotypes: IgG1, IgG2a (or IgG2c), IgG2b, and IgG3. In humans, there are four IgG isotypes: IgG1, IgG2, IgG3, and IgG4, and two IgA isotypes: IgA1 and IgA2. Class switching to different immunoglobulin isotypes is regulated by B cell activation and cytokines (19–22). Class switch recombination to IgE is dependent on IL-4/IL-13 signaling through IL-4Rα and STAT6. In humans, IgE production is usually associated with IgG1 and IgG4 production (23, 24), and in mice, with IgG1 production (25). In terms of function, IgM and IgG antibodies are important for neutralization and clearance of pathogens through complement fixation and binding to receptors of phagocytes (18). IgA antibodies are transported through the epithelium to luminal cavities such as the gut, where they regulate pathogens and maintain homeostasis with the microbiota (18). The main biological activity of IgE derives from its ability to bind in monomeric form to high affinity FcεRI receptors on mast cells and basophils, inducing their degranulation upon crosslinking caused by antigen binding (26). IgE also binds to FcεRII (CD23) on FDC and B cells, regulating antigen presentation and IgE production (1, 27–29).

The combination of class switch recombination, affinity maturation and cell differentiation results in the generation of class-switched memory B cells and long-lived plasma cells that are the essential components of effective B cell memory (30–32).

### Memory B Cells

Memory B cells are antigen-experienced quiescent B cells (33) that respond rapidly and vigorously to activation, generating a humoral response that is of higher magnitude and affinity than the primary response (31, 32, 34). Memory B cell survival is independent of the presence of cognate antigen (35).

Memory B cells represent a heterogeneous population. They differ in their origin, immunoglobulin isotype, mutation rates, and differentiation potential. Memory B cells may originate from within or outside of germinal centers. Extrafollicular memory cells are typically generated early in an immune response and carry low number or no somatic mutations. Germinal center-derived memory cells are continuously generated during a germinal center response, comprising clones with diverse affinities. Memory B cells may express IgM or switched immunoglobulin isotypes, and they may be programmed to differentiate into either plasma cells or germinal center cells upon activation (31, 32, 34). IgM memory cells typically have fewer somatic mutations than IgG memory cells, and preferentially become germinal center cells in secondary responses, while plasma cells are typically generated from IgG memory cells (13, 36).

The propensity for IgG memory cells to preferentially and rapidly differentiate to plasma cells has been attributed to increased intracellular signaling of the IgG BCR compared with the IgM BCR (30). There are however other cell-intrinsic factors that affect the differentiation potential of IgG1 memory cells, and the full diversity of the IgG1 memory population has only recently started to be appreciated (14, 37). Subsets of secondary lymphoid organ memory IgG1 cells that preferentially give rise to plasma cells or germinal center cells after activation in secondary responses were identified in mice (14, 37). These subsets, which we called pro-plasma cell (pro-PC) and pro-germinal center cells (pro-GC), respectively, express distinct transcriptional programs and respond to activation with different kinetics (14). Importantly, the pro-PC subset, which expresses CD80 and CD73, is responsible for the rapid generation of plasma cells secreting high affinity antibodies in memory responses. Transcriptional analysis showed that pro-PC IgG1 memory cells express *Zbtb20*, a transcription factor that promotes plasma cell differentiation, while pro-GC IgG1 memory cells express *Bcl6, Foxp1, Foxo1*, and *Bach2*, transcription factors that inhibit plasma cell formation while promoting the germinal center fate (14). Thus, the differentiation potential of pro-PC and pro-GC memory cells is hardwired though the expression of distinct transcription factors. The specific roles of the IgG1 memory subsets in the generation of IgE plasma cells is discussed in section IgG1 Memory Cells are Precursors of IgE Plasma Cells. A lung resident memory B cell population which is phenotypically different from memory B cells of spleen and LN was recently described in mice infected with influenza virus. This tissue resident B memory population contributes to rapid local plasmablast differentiation following intranasal challenge (38).

Human memory B cell subsets have also been identified among unswitched (IgM^+^) and switched B cells, but their correspondence to mouse memory B cell subsets is not yet clear. Human memory B cells that differ in CD27 expression have been described. CD27^+^ human memory B cells have somatically mutated BCR genes, while CD27^−^ memory B cells are mostly unmutated or carry a lower number of mutations than CD27^+^ memory cells (39–42). The mutation frequency and replication history of memory B cells suggest that CD27^+^ memory cells are derived from germinal center responses, while CD27^−^ memory cells form outside germinal centers (40). Human memory cells expressing CD27 and CD80 can efficiently activate T cells and differentiate into plasma cells that secrete class-switched antibodies (43). Thus, in both human and mouse, CD80 expression marks memory B cells with the ability to become plasma cells. CD80 may be functionally important in this process, as CD80-deficient B cells produced fewer antigen-specific plasma cells in spleen and bone marrow after immunization (44).

The life span of memory B cells varies greatly. Some human memory B cells can be detected for decades, as in the case of smallpox-specific IgG memory cells (45). In other cases, such as in the B cell memory response to malaria merozoite (46) and to some epitopes of influenza virus (47), the memory B cell population declines quickly after infection. Though the factors that determine memory B cell longevity are still largely unknown, recent work in mice has shown that the frequency and affinity of antigen-specific naïve B cells play a role (48). The longevity of memory cells could be important in the context of allergy, as it may explain why individuals can remain allergic for years following their last encounter with allergen. However, this allergic memory could also be maintained by long-lived IgE plasma cells.

### Long-Lived Plasma Cells

Much of our understanding of the dynamics of plasma cell generation derives from mouse studies. Primary immune responses lead to an initial wave of short-lived plasma cells (49, 50) that provide an important source of early, low-affinity antibodies. Additionally, in most primary responses, other activated B cells form germinal centers, where they proliferate extensively and undergo affinity maturation. The early germinal center primarily generates memory cells, with increasing plasma cell output over time (51).

The germinal center cells that will differentiate into plasma cells are among the pool of high affinity clones (12, 52, 53). Similarly, in memory responses, high affinity plasma cells rapidly differentiate within a week after challenge from clonal expansion of a subset of specialized high affinity memory cells (14). Newly formed plasma cells divide in the secondary lymphoid organs, then enter the circulation to migrate to the bone marrow, where they complete the process of terminal differentiation into non-dividing, long-lived plasma cells that secrete large amounts of antibody (6, 7). Long-lived IgG plasma cells are predominantly located in the bone marrow, but they are also present in the spleen, albeit in lower numbers (7, 29). IgA plasma cells form mainly in the intestine and home to the intestinal lamina propria (54–56), though they also can be found in the bone marrow and the spleen (57). Plasma cells secrete immunoglobulin at rates estimated to reach 10,000 molecules per second (7).

The differentiation of B cells into plasma cells requires a profound cellular reprograming (58). The prototypical B cell pathways including BCR signaling, and antigen processing and presentation are downregulated; while pathways involved in protein synthesis, N-glycosylation, endoplasmic reticulum (ER) stress and the unfolded protein response are upregulated (59–62). The ER and Golgi systems expand substantially, and metabolic reprogramming supports the secretory demands of the plasma cell: lipid synthesis increases to accommodate organelle remodeling, while glucose uptake and oxidative phosphorylation are upregulated to fuel plasma cell function (61, 63, 64).

The longevity of plasma cells can span from a few days to decades. Since bone marrow plasma cells die rapidly *ex vivo*, plasma cell lifespan is likely determined by the anatomical microenvironment, notably the specialized bone marrow niche that is believed to sustain long-term plasma cell survival (65–67). In this niche, plasma cells physically interact with stromal cells and hematopoietic cells that secrete factors important for plasma cells retention and survival within the niche, including CXCL12, APRIL, BAFF, and IL-6. Bone marrow stromal cells support plasma cell survival *in vitro* through VLA4-VCAM interactions and IL-6 production (68). In the bone marrow, plasma cells localize adjacent to VCAM-1^+^ stromal cells that produce CXCL12 (69). Plasma cells that lack CXCR4, the receptor for CXCL12, mis localize in the spleen, accumulate in circulation, and fail to home to the bone marrow (70). Among hematopoietic cells, eosinophils, basophils, and megakaryocytes contribute to plasma cell survival by producing APRIL and IL-6 (71–73). Plasma cells deficient in BCMA, the receptor for APRIL and BAFF, have impaired survival in the bone marrow (74), and both APRIL and BAFF support plasma cell survival *in vivo* (75). The evidence for reliance on other cell types strongly supports an important role for cell-extrinsic factors in plasma cell longevity.

It is unclear to what extent plasma cell longevity is also affected by cell-intrinsic factors. Several pro-survival genes in the *Bcl-2* family are expressed at higher levels in plasma cells than in other B cells, and plasma cell expression of the anti-apoptotic gene *Mcl-1* is required for survival beyond a few weeks *in vivo* (76). However, *Mcl-1* expression is itself regulated by BCMA (76), the receptor for APRIL and BAFF - both cell-extrinsic survival factors. Recent work has revealed metabolic differences between splenic plasma cells at day 7 post-immunization, which are enriched in short-lived plasma cells, compared with the more typically long-lived plasma cells in bone marrow (77). Bone marrow plasma cells were shown to uptake more glucose, import more pyruvate into mitochondria, and adapt better to bioenergetic pressure than splenic plasma cells, suggesting that these differences contribute to their long-term survival (77).

Long-lived plasma cells are an essential component of immunity whose function is to continuously secrete antibodies. Long-lived plasma cells originate from germinal center reactions, and home to bone marrow niches that support their survival. Questions remain on the immune conditions that allow differentiation of long-lived plasma cells, and the relative contribution of cell-intrinsic and niche factors to plasma cell survival and longevity. IgE plasma cells have not yet been thoroughly studied, and have only recently received more attention. They are discussed in detail for mice in section Most IgE Cells are Plasma Cells, and for humans in section Human IgE Cells.

## The IgE Memory Response in Mice

There is strong evidence that IgE responses have memory. Secondary IgE responses to helminth infection and to immunization in mice are faster and of greater magnitude than the primary response (78, 79), which is typical of B cell memory. Consistent with B cell memory, the affinity of IgE antibodies and the frequency of high affinity mutations in IgE genes increase with repeated immunization (14, 80–83). Paradoxically, there are many hurdles for IgE memory: the IgE germinal center phase is exceptionally transient, and there is a paucity of bona fide IgE memory cells (14, 80, 81, 83).

A number of studies have provided strong evidence that the memory for IgE responses depends on IgG1 memory cells that class switch and differentiate to IgE plasma cells (14, 82, 84, 85). This mechanism compensates for the paucity of true IgE memory cells while at the same time imposing great stringency to IgE production in memory responses, as T cell help and high levels of IL-4 are required for *de novo* switching to IgE (84). The next sections will discuss the current knowledge of how IgE memory responses in mice are generated and maintained.

### IgE Germinal Center Cells and the Missing IgE Memory Cells

The identification of IgE germinal center cells in mice has for a long time been hampered by the transient nature of this population, and by their very low expression of membrane IgE. The development of fluorescent protein IgE-reporter mice (81, 83), and improved labeling methods using the anti-IgE monoclonal antibody R1E4 (81, 84), which does not recognize IgE bound to cellular FcεRI or FcεRII (86, 87), have facilitated the functional analysis of live IgE-expressing cells.

**Figure 3:**
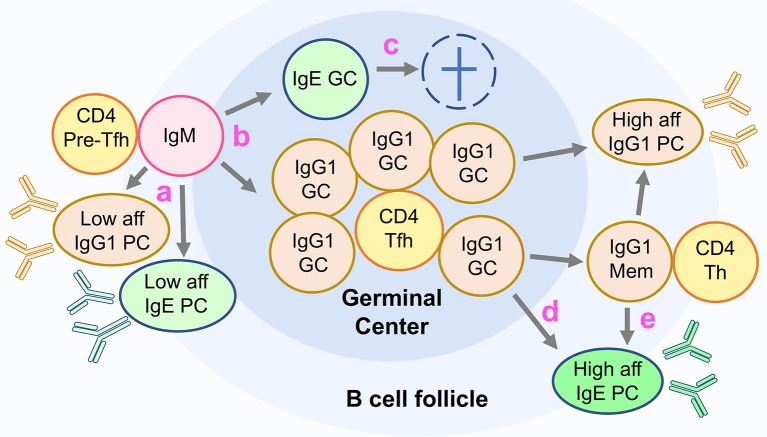
Unconventional differentiation of IgE cells in mice. The figure depicts the current model of IgE cell differentiation in mice. (a) In primary responses, activated IgM expressing lymphocytes undergo class switch recombination to IgG1 and IgE producing a fast wave of low affinity short lived plasma cells (PC). (b) Some activated IgM cells switch to IgG1 or IgE and differentiate into germinal center cells (GC). (c) IgE GC cells are transient and do not generate IgE memory cells or high-affinity IgE PC. (d) High affinity IgE PC are generated in primary responses from IgG1 GC cells, and (e) in memory responses from IgG1 memory cells.

IgE and IgG1 germinal center cells form early in primary responses (81, 83), coinciding with the peak of IL-4 production (88). Unlike IgG1 germinal center cells that persist from several weeks to months, IgE germinal center cells quickly disappear during the primary response, declining rapidly from a peak at day 10–12 post primary immunization, and are very scarce in secondary responses accounting for <0.1% of B220^+^CD138^−^ cells (14, 81, 83) (Figure 3).

Compared to IgG1 germinal center cells, IgE germinal center cells have several deficits that impair their function: they have a two- to threefold higher rate of apoptosis, express the BCR at three- to fourfold lower levels and have overall reduced intracellular BCR signaling (81). In addition, IgE germinal center cells have decreased cell membrane expression of important co-stimulatory molecules CD21/35, OX-40L and ICOSL, and are markedly underrepresented in the light zone of the germinal center. While approximately one in three IgG1 germinal center cells are light zone cells, one in seven IgE germinal center cells are light zone cells (81). These findings suggest impaired IgE function in the light zone of the germinal center, where newly mutated germinal center cells typically test their BCR through interactions with FDC and Tfh cells (8).

We investigated if the low expression of the BCR in IgE germinal center cells was due to decreased amount of heavy chain transcripts. We found that the overall heavy chain transcript level was the same between IgG1 and IgE germinal center cells. However, there was a difference in the ratio of membrane to secreted IgE transcripts in IgE germinal center cells, with a predominance of secreted IgE transcripts (81). Three atypical weak polyadenylation signals (AGTAA, AAGAAA, and ATTAAA) are found downstream of IgE membrane exons in human and mouse IgE genes, while an optimal consensus AATAAA sequence signals the polyadenylation of secreted IgE transcripts (89). The weak polyadenylation signals for membrane IgE favor the production of secreted IgE in B220^+^IgE cells (89) and likely in IgE germinal center cells (81).

The transient nature of IgE germinal center cells may be responsible for the paucity of true functional IgE memory cells in mice (14, 81, 83). It is also possible that IgE memory B cells form but die very rapidly without contributing to memory responses.

### Most IgE Cells Are Plasma Cells

The predominance of the plasma cell fate is one of the hallmarks of the IgE response (80). Various factors may contribute to this: the transient and apoptotic IgE germinal center phase, the lack of IgE memory cells, and a tendency for IgE B cells to differentiate into plasma cells (Figure 3).

Intriguingly, recent work suggests that the very expression of the IgE BCR intrinsically drives plasma cell differentiation independently of antigen binding (30, 90–92). B cells expressing membrane IgE relocated the IgE BCR to lipid rafts, changed cell morphology, migrated less to CXCL12 and underwent increased apoptosis (91). Ectopic expression of membrane IgE in primary B cells *in vitro* resulted in increased autonomous BCR signaling that triggered plasma cell differentiation (90, 92). This increased BCR signaling could be attributed partly to a physical association of membrane IgE with CD19. Accordingly, IgE plasma cell differentiation *in vitro* was suppressed by reduced BCR signaling in CD19- and Blnk-deficient B cells. *In vivo*, reduced BCR signaling by CD19 haplo-insufficiency or Blnk deficiency increased the IgE germinal center cell population at the expense of IgE plasma cell population (83, 90). A different experimental approach compared B cells engineered to express the heavy chain of IgG1 (*Igh*γ*1*) or IgE (*Igh*ε) from development (93). *Igh*ε*/*ε mice had low numbers of mature B cells, and these B cells expressed BCR at low levels, similarly to what has been described for germinal center IgE cells. Low IgE BCR expression and signaling may be responsible for the inability of IgE to promote normal B cell development. Augmenting BCR signaling through PTEN deletion rescued mature B cell numbers in the *Igh*ε*/*ε mice. Thus, while overexpression of membrane IgE *in vitro* promotes plasma cell differentiation through increased tonic BCR signaling, both IgE germinal center cells and naïve B cells expressing membrane IgE have lower BCR density, decreased BCR signaling and are highly apoptotic. The *in vivo* conditions in which membrane IgE signaling promotes plasma cell differentiation remain undetermined, but membrane IgE expression is essential for IgE responses, as deletion of IgE membrane exons in mice leads to an almost complete absence of IgE production *in vivo* (78).

A distinct characteristic of mouse IgE plasma cells is that they express higher levels of membrane immunoglobulin than IgE germinal center cells (80, 81, 83) and IgG1 plasma cells. Whether membrane IgE is important for the survival of IgE plasma cells and whether it could have signaling function in mice is not known. BCR signaling function has been described for IgM and IgA plasma cells (94, 95), so it is plausible that such function could occur in IgE plasma cells.

Mouse IgE plasma cells are also unusual in that they accumulate in secondary lymphoid organs, where they may constitute one third or more of all plasma cells, and have a delayed accumulation in the bone marrow (80, 81, 83). When equal numbers of IgG1 and IgE plasma cells isolated from lymphoid organs of immunized mice were transferred intravenously to naïve mice, serum IgG1 titers were much higher than those of IgE. While transferred IgG1 plasma cells localized mostly to the bone marrow, IgE plasma cells could be found in the spleen and bone marrow (81). IgE plasma cells appear thus to be less efficient than IgG1 plasma cells in homing to the bone marrow, and whether directly related, in contributing to the circulating antibody pool. Increased IgE plasma cell apoptosis and decreased responsiveness to CXCL12 (91, 96) may be some of the factors involved in the poor bone marrow localization of IgE plasma cells.

#### The Life Span of IgE Plasma Cells

A very important question for the understanding of allergic responses is whether long-lived IgE plasma cells exist. Experimental evidence in mice has demonstrated the existence of long-lived and short-lived IgE antibody responses. Early experiments in the 1980s showed that IgE-secreting cells and IgE antibodies were present for up to a year after intraperitoneal immunization of mice and rats with purified protein in alum adjuvant. A large part of the IgE response remained after lethal X-ray irradiation, a treatment that depletes memory cells but not plasma cells (97–99). In a more recent study, mice immunized intraperitoneally with OVA in alum and then re-challenged by OVA inhalation had IgE, IgG, and IgA plasma cells in lung, spleen and bone marrow. Like their IgG and IgA counterparts, IgE plasma cells disappeared from the lung after termination of OVA inhalation, but persisted in spleen and BM until the end of the analysis at day 100 after immunization. Spleen and BM plasma cells were resistant to cyclophosphamide, a drug that depletes dividing plasmablasts but not plasma cells, suggesting that plasma cells in these compartments are long-lived (100).

Further evidence for the persistence of IgE antibodies and anaphylactic responses was obtained by prolonged treatment of peanut-immunized mice with anti-CD20 antibodies, which deplete naïve and memory B cells but not plasma cells. Serum IgE antibodies and IgE plasma cells were not affected by a 15-week treatment with anti-CD20 antibodies (101).

Treatment with the proteasome inhibitor bortezomib to deplete IgE plasma cells has provided more ambiguous results, in one case reducing circulating IgE and plasma cells but not allergy (102), and in another two studies, reducing IgE and suppressing allergic reactivity (103, 104). The variable results described in studies targeting plasma cells with bortezomib could be due to different sensitivities of plasma cells populations, and to an effect of plasma cells rebounding after plasma cell depletion (105). The persistence for several weeks of IgE bound to mast cells may also explain allergic reactivity in the absence of detectable serum IgE.

A recent study using a model of oral sensitization to peanut characterized the anaphylactic response and antigen-specific IgE and IgG1 levels during 15 months after sensitization (103). While peanut-specific IgG1 antibodies persisted during this period, specific IgE antibodies were undetectable at 6 months after sensitization, a time that coincided with a large decrease in the anaphylactic response. In this model, the authors calculated the half-life of IgG1 plasma cells to be 234 days and that of IgE plasma cells to be only a quarter of that, or 60 days. By transferring serum of sensitized mice to IgE-deficient naïve mice and measuring their anaphylactic response, they estimated that the half-life of IgE bound to mast cells was 67 days, compatible with previous findings (106).

Taken together, these studies indicate that IgE plasma cells can persist in mice from a few months to a year in the absence of a new allergen exposure. Although the precise determinants of plasma cell longevity in general are under debate (section Memory B Cells), the unique factors affecting IgE plasma cell longevity are even less clear. Recent efforts have focused on describing the lifespan of IgE plasma cells and identifying factors that impact the generation of IgE plasma cells.

### The Role of IgG1 Memory B Cells as Precursors of IgE Plasma Cells

Sequencing of the switch (S) region of mouse and human IgE heavy chain genes showed that the junctional Sμ-Sε sequences often contained remnants of IgG switch regions (Sμ-Sγ-Sε), specifically Sγ1 in mice, and Sγ1 and Sγ4 in humans (25, 87, 107–109). The concept of sequential immunoglobulin class switching was thus established, whereby a B cell will undergo a switch from IgM to a downstream isotype, usually IgG (generating an Sμ-Sγ hybrid S region), followed by a second sequential switch, to IgE (generating a Sμ-Sγ-Sε hybrid S region) (Figure 2). The biological significance of sequential switching in IgE responses was only appreciated much later. As will be discussed below, we found that sequential switching from IgG1 to IgE is essential for the generation of high affinity IgE.

Our early studies of IgE cell biology showed a predominance of IgE plasma cells, a paucity of IgE germinal center cells, and an absence of IgE memory cells (80). Nevertheless, the presence of mutations in the BCR variable region indicated that IgE antibodies underwent affinity maturation. We proposed at the time that maturation of the IgE antibody response occurred in a precursor IgG1 cell phase, and we showed that purified IgG1 cells could undergo class switching to IgE *in vivo* and *in vitro* (80). To determine if sequential switching was necessary to produce high affinity IgE, we analyzed the IgE response in mice genetically deficient in class switching to IgG1 (82). IgG1-deficient mice, in which IgE cells were generated by direct switching from IgM cells, produced IgE at levels comparable to wild type mice after repeated immunization, but were unable to efficiently generate affinity matured IgE (82). Furthermore, we showed that IgM^−^IgD^−^B220^+^ switched B cells (containing germinal center and memory B220^+^ IgG1^+^ and IgE^+^ cells) generated a recall IgE response identical to IgE-depleted switched B cells (81). These findings demonstrate that B220^+^ IgE cells do not contribute to the formation of high affinity IgE plasma cells and to the memory of IgE responses. Instead, these results indicate that these functions are contained in the IgG1 population (Figure 3).

The study of switch regions in DNA of IgE germinal center and IgE plasma cells shed light on the developmental origin of these populations. We found that IgE germinal center switch regions did not contain Sγ1 remnants while in contrast a substantial portion of IgE plasma cells did. The percentage of IgE plasma cell switch regions with Sγ1 remnants increased after repeated immunization to approximately 60%, and was highest in the bone marrow (81, 82). These results indicate that in mice, IgE germinal center cells differentiate directly from IgM cells, whereas a substantial number of IgE plasma cells originate from IgG1 cells (81). Since Sγ1 remnants may be lost in the second recombination process, the proportion of IgE plasma cells that derive from IgG1 precursors is likely to be even higher. These findings are consistent with the concept of IgE plasma cells deriving principally from IgG1 germinal center cells or from IgG1 memory cells, rather than from IgE germinal center cells.

The Voehringer group studied the B cell repertoire in murine helminth infection, and found considerable overlap between the repertoires of IgG1 and IgE cells, indicating a common precursor origin of these two isotypes (85). Furthermore, they show that an IgE memory response could be generated by adoptive transfer of IgG1 cells (85). These results demonstrate a fundamental role for IgG1-expressing cells in the memory of IgE responses.

The findings described above are consistent with previous observations that IL-4, a cytokine necessary for class switching to IgE, is required for the production of IgE but not IgG1 in secondary responses (110). This indicates that IgE production in memory responses involves *de novo* class switching to IgE, rather than the activation of supposed IgE memory cells.

**Figure 4:**
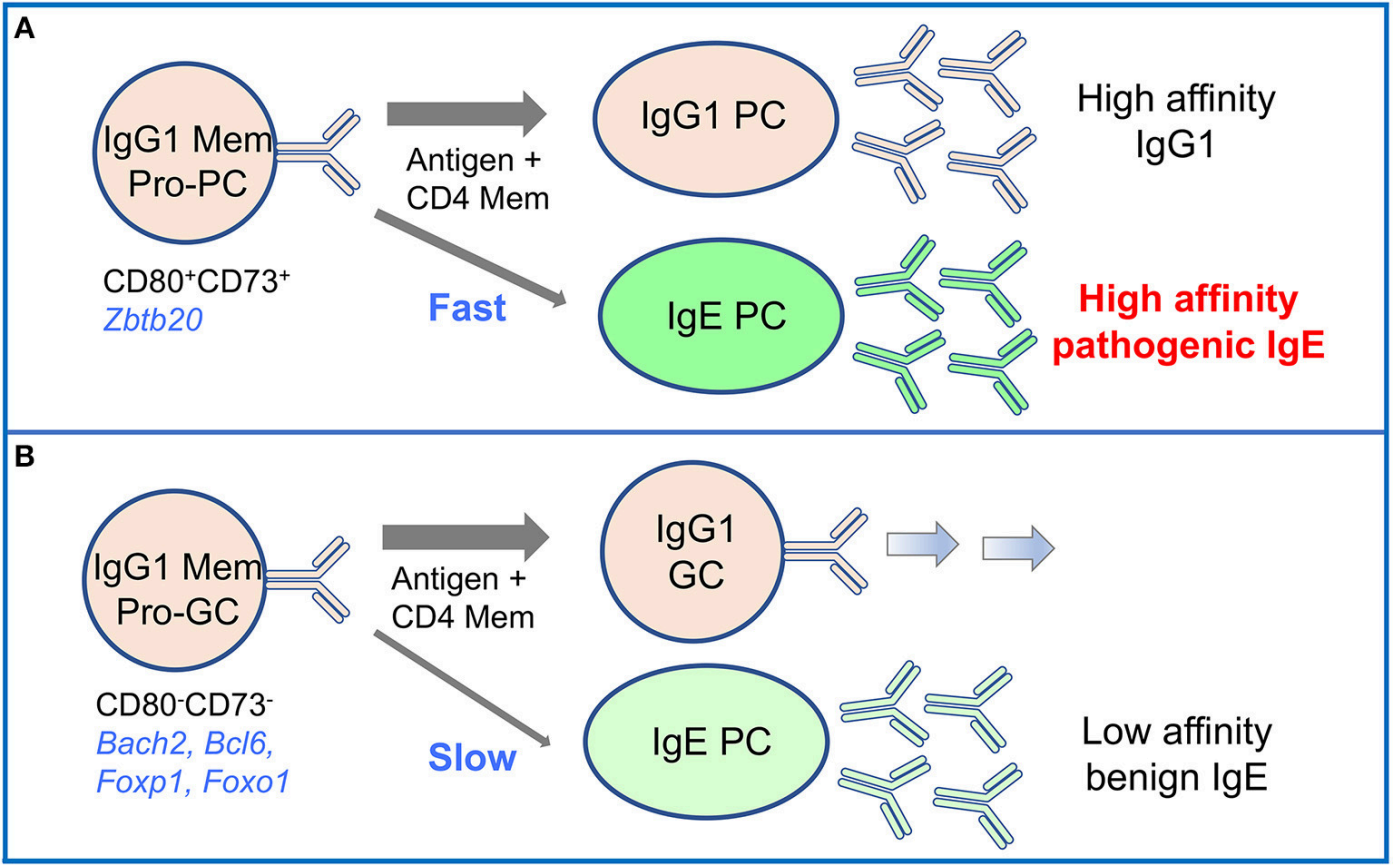
High-affinity IgE is derived from the CD80^+^ pro-PC subset of IgG1 memory cells in mouse memory responses. Two subsets of mouse IgG1 memory cells identified by differential expression of CD80 and CD73 have distinct differentiation potential upon activation. **(A)** CD80^+^CD73^+^ pro-plasma cell (Pro-PC) IgG1 memory cells generate with fast kinetics IgG1 PC and IgE PC enriched in high affinity clones. IgE antibodies derived in this pathway mediate anaphylactic responses. **(B)** CD80^−^CD73^−^ pro-germinal center (Pro-GC) IgG1 memory cells respond to activation with slower kinetics. They preferentially differentiate into IgG1 GC cells, and also generate a late response of low affinity non-pathogenic IgE PC.

We have more recently investigated the role of different subsets of IgG1 memory cells in the IgE memory response (14). As described above (see section Memory B Cells), we identified pro-PC and pro-GC subsets of IgG1 memory cells that preferentially differentiate into IgG1 plasma cells or IgG1 germinal center cells, respectively. We found that the pro-PC IgG1 memory subset give rise to IgE plasma cells secreting high affinity IgE antibodies capable of mediating anaphylaxis (Figure 4). Thus, the pro-PC IgG1 memory population contains a memory for allergic responses that under conditions involving T cell help and IL-4 signaling can be activated to generate IgE plasma cells by *de novo* class switching to IgE (14).

Interestingly, the pro-GC IgG1 memory subset also gave rise to IgE plasma cells, but this IgE response was delayed and produced IgE plasma cells secreting low affinity IgE (Figure 4). This is intriguing, as high affinity clones were also present among the pro-GC IgG1 memory population, albeit at an approximate two-fold lower frequency than in the pro-PC population, hinting at distinct mechanisms of clonal selection operating in these two memory subsets (14).

An important observation of this study is that the vast majority of IgE cells generated from both IgG1 memory cell subsets were IgE plasma cells, with almost no IgE germinal center or memory cells being produced. This is consistent with previous findings of Sγ1 remnants in the switch regions of IgE plasma cells but not in IgE germinal center cells, which led to the conclusion that IgE germinal center cells are generated directly from IgM cells, while sequential switching from IgG1 cells only generates IgE plasma cells (81).

The differentiation potential of IgG1 memory subsets manifests differently in IgG1 and IgE progeny. IgG1 pro-PC and IgG1 pro-GC memory subsets guide their fate into IgG1 plasma cells or IgG1 germinal center cells, respectively, in a largely pre-determined B cell autonomous way. In contrast, their differentiation into IgE plasma cells is likely determined by the expression of the new IgE BCR after class switching to IgE. It is possible that IgE BCR signaling contributes to the formation of IgE plasma cells (90, 92) from IgG1 memory cells regardless of precursor IgG1 subset.

In sum, several lines of evidence obtained by various groups strongly supports an essential role for IgG1 memory cells in the generation of IgE plasma cells.

## The Human IgE Response

While increasing evidence has accumulated on the generation and memory of IgE responses in mice, comparably much less is known about human IgE cell differentiation and memory. Much of the current thinking has been influenced by the experimental studies in mice. The characteristics of human IgE plasma cells and whether bona fide human IgE memory cells exist remain largely unknown. Nevertheless, a number of studies of human IgE cells *in vivo* and *in vitro*, as well as several longitudinal studies on the development of IgE and IgG antibodies, provide insights into the mechanisms of human IgE cell differentiation and memory in atopic patients, and these will be discussed in the following sections.

### Human IgE Cells

In humans, the extremely low frequency of IgE cells, together with the “false positives” which derive from the stable and prolonged binding of monomeric IgE to basophils and other cells in blood, greatly hindered the study of human IgE cells. Nevertheless, human IgE plasma cells were first identified in peripheral blood of atopic and normal subjects as IgE-secreting cells by ELISPOT (111), and then by flow cytometry analysis of CD138^+^ purified cellular fractions (112). IgE plasma cell frequency correlated with the concentration of serum IgE and with the amount of IgE produced spontaneously in cell culture. Interestingly, and in sharp contrast to mice, human IgE plasma cells were found to express low levels of membrane IgE (112). The differentiation of IgE plasma cells in the respiratory mucosa of allergic patients by direct and sequential switching was demonstrated through the identification and sequencing of switch products (113). IgE production has also been demonstrated in cultures containing respiratory mucosal explants (107, 113–115).

Although there is no direct evidence illustrating the process of human IgE cell differentiation *in vivo*, the origin of IgE cells can be partially inferred from the analysis of somatic mutations, switch recombination products, and antigen receptor repertoire, which may shed light on the memory cell subsets from which human IgE plasma cells differentiate.

Recently, the van Zelm group characterized IgE plasma cells and IgE memory cells in atopic and non-atopic subjects (116, 117). They analyzed the phenotype, replication history, and mutation rates of IgE cells in human blood and tonsil samples. They identified IgE plasma cells by CD19 and CD38 expression (CD19^+^CD38^hi^), but these IgE plasma cells did not express CD138. This description of IgE plasma cells contrasts with the one by Horst and collaborators described above (112). IgE plasma cells and IgE memory cells (characterized as CD19^+^CD38^dim^) were increased in the blood of children with atopic diseases (117). The frequency of somatic mutations in IgE plasma cells was similar to IgM plasma cells and lower than in IgG or IgA plasma cells. IgE memory cells could be further subdivided by CD27 expression. IgE^+^CD27^+^ memory cells had more mutations, higher replication history, and higher frequency of Sγ1 footprints in their switch regions than the IgE^+^CD27^−^ memory cells, indicating that IgE^+^CD27^+^ cells are more likely to have an IgG1 germinal center origin. Both IgE^+^CD27^+^ and IgE^+^CD27^−^ memory cells were atypical compared to other memory B cells: they expressed very low levels of the BCR signaling molecule CD79b (Igβ), and low levels of membrane immunoglobulin (116). Although these human IgE memory cells were atypical in their low BCR expression, they could differentiate into plasma cells *in vitro* (116). These findings suggest potential differences between mice and humans regarding the existence of IgE memory cells. However, given that recall responses of memory B cells depend on BCR recognition and signaling, it is unclear how adequately these abnormal IgE B cells can exert memory functions *in vivo*.

In contrast, and in support of IgE-expressing cells in humans being predominantly plasma cells, a recent study showed that circulating IgE-expressing cells in food allergic individuals were mostly plasmablasts with immature phenotype (118). Sequencing of the variable regions of single cell sorted IgE cells identified a clonal family with identical gene rearrangements in unrelated individuals. By cloning and expressing six IgE antibodies belonging to the one shared clonal family the authors showed that these antibodies had strikingly high affinity for peanut antigen Ara h2, with cross-reactivity to other peanut antigens (118). These studies propose important potential mechanisms of clonal selection and affinity maturation of human IgE cells. Further work is necessary to determine the generality of these findings in atopic conditions and in healthy individuals.

To elucidate the ontogeny of human IgE cells, Ramadani and collaborators compared the ability of tonsillar B cell populations to generate IgE cells after stimulation with anti-CD40 antibodies and IL-4 (119, 120). Among tonsillar B cells, the IgE cells generated from germinal center cells had the highest frequency of Sγ remnants indicative of sequential switching (120), and these germinal center cells generated more IgE cells than naïve or memory cells. IgE plasmablasts and plasma cells, and IgE cells with characteristics of germinal center cells were identified in this culture system. Total tonsil B cells gave rise to IgE plasma cells by direct and sequential switching. Purified tonsil germinal center B cells generated IgE plasma cells by sequential switching, and these were the main source of IgE plasma cells. As in mice (84), more IgE cells than IgG1 cells had a plasma cell phenotype.

The membrane form of human and primate IgE, but not murine IgE, can be expressed as two different isoforms generated by alternative splicing (121). The long isoform contains an additional extracellular membrane-proximal domain (EMPD) of 52 amino acids. The long isoform was found to be transported to the plasma membrane at a slower rate than the short isoform (121), and to regulate survival and signaling in transfected mouse B cell lines (122). The two membrane isoforms of IgE have been detected *ex vivo* in human cells (30). Recent studies using retroviral transduction of a human B cell line demonstrated that the short isoform was abundant in the plasma membrane, while the long form localized mainly to the endoplasmic reticulum (ER) (123). Both isoforms of membrane IgE assembled with the BCR signaling complex Igα/Igβ, but intracellular signaling was diminished in cells expressing the long isoform. Interestingly, IgE plasma cells generated *in vitro* from tonsillar B cells downregulated the expression of the short isoform of membrane IgE while upregulating the expression of the EMPD-containing long isoform of IgE (119).

In summary, IgE plasma cells and IgE memory cells have been identified in human blood and tonsils, and germinal center-like IgE cells and IgE plasma cells have been generated *in vitro*. There is ample evidence that at least part of the human IgE cell population is generated through the sequential switching of IgG cells, and that the sequential switching and germinal center origins are associated. The existence of an *in vivo* functional population of human IgE memory remains unproved, while new data supports the predominance of the plasmablast phenotype among circulating IgE cells. Furthermore, it remains unclear whether a bona fide population of IgE germinal cells is present in humans, and whether it shares a similar pro-apoptotic phenotype as in mice.

### Life-Span of Human Serum IgE Responses

Human IgE responses are diverse in magnitude, localization, and in the clinical symptoms that manifest. For example, IgE production and IgE-mediated mast cell reactivity may be mainly localized to the nasal mucosa, with low systemic impact. In other cases, widespread mast cell sensitization and systemic reactions may occur, as is the case with IgE-mediated anaphylaxis. Furthermore, IgE responses may be regulated periodically by variations in allergen exposure resulting in generation of short-lived IgE plasma cells, or may be sustained over time, either because of chronic exposure to perennial allergens, or due to long-lived IgE plasma cells. As discussed below, there is evidence for both short-lived and long-lived humoral IgE responses.

An example of seasonal variation in circulating IgE was shown in a 3-year follow up of a patient allergic to grass pollen. The seasonal increase in pollen count was immediately followed by a temporary serum increase of pollen-specific IgE and IgG4 (124). This finding demonstrates the existence of a memory response to pollen, and suggests that relatively short-lived IgE plasma cells periodically formed in the pollen season. Another study, in contrast, provides evidence of the existence of long-lived human IgE plasma cells. When patients that had been infected with filaria moved away from the endemic area and were treated with anti-helminthic therapy, they continued to have anti-filaria IgE antibodies several years after treatment, though at lower levels than before treatment (125). Newly acquired allergies to food and environmental allergens in patients that underwent allogeneic bone marrow transplantation (126–128) have been considered evidence of long-lived IgE plasma cells. While this could be the case, it is also important to consider that the bone marrow contains T and B memory cells, and these could have been responsible for the generation of allergen-specific IgE plasma cells in the transplanted individuals.

The contribution to serum IgE of long-lived IgE plasma cells vs. continuously generated short-lived IgE plasma cells could be evaluated if the production of new IgE plasma cells was inhibited. If, as suggested in some mouse and human studies, formation of new human IgE plasma cells in sensitized patients requires *de novo* class switching to IgE (rather than differentiation from true IgE memory cells), inhibiting class switching to IgE should greatly reduce the formation of new IgE plasma cells. Since class switching to IgE in humans depends on the cytokines IL-4 and IL-13, some inferences can be drawn from the treatment of atopic patients with dupilumab, an anti-IL-4Rα antibody that inhibits IL-4 and IL-13 signaling. Serum IgE levels were reduced by about 40–50% after 3–4 months of treatment of patients with asthma (129), atopic dermatitis (130), and chronic rhinitis (131). This suggests that at least part of the serum IgE pool is derived from continuous generation of new IgE plasma cells that survive only few months. As a proof that *de novo* class switching to IgE was prevented by blocking IL-4Rα signaling, patients with ongoing dupilumab treatment did not produce IgE to new vaccine antigens (132). While the dupilumab treatment suggests continuous *de novo* production of IgE plasma cells in atopic patients, it is important to point out that the reduction of serum IgE was only partial and serum IgE levels remained quite high at the end of the treatment. Thus, the existence of very long-lived IgE plasma cells in atopic patients cannot be excluded. It remains to be seen if IgE can be further reduced by longer dupilumab treatment, if allergen specific-IgE levels can decrease to the point of demonstrating prevention of allergen-driven mast cell degranulation, and how stable the changes in IgE levels are when treatment is discontinued.

The findings described above illustrate the variation in the persistence of serum IgE in different atopic conditions, which are compatible with both short-lived and long-lived human IgE plasma cells. As for other plasma cells, the microenvironment for generation and homing of IgE plasma cells may affect their long-term survival.

### Is Human IgE Memory Contained in IgG Memory Cells?

Several lines of evidence are compatible with the notion that human IgG memory cells are the predominant precursors of IgE plasma cells, during allergic sensitization and in allergic memory responses. These findings derive from studies on the development and association of specific IgG and IgE responses in children and adults (Table 2), on the analysis of the switch-recombination history of IgE genes, and on the relatedness of the IgE and IgG immunoglobulin repertoires.

**Table 2:** Development of allergen-specific IgG antibodies is generally associated with allergen-specific IgE sensitization.

**Study population age**	**Allergens**	**Findings**	**+ Association between IgG and IgE**	**References**
3–60 months	Food allergens: chicken ovalbumin, cow's milk beta-lactoglobulin Airborne allergens: mite and rye.	Specific IgG antibodies were detected before the appearance of specific IgE antibodies.	Yes	(133)
3 months to 8 years	Food and airborne allergens	At-risk children with high IgG against food allergens, were more likely to develop IgE antibodies against airborne allergens.	Yes	(19)
6, 18 months, and 8 years	Food allergen: ovalbumin. Airborne allergens: pollen Bet v 1 and cat dander.	Positive correlation between IgE sensitization, clinical allergy and high levels of specific IgG1 and IgG4 antibodies.	Yes	(23)
1 and 6 years	Food and airborne allergens.	Increased IgA and IgG antibodies against gliadin or cow's milk b-lactoglobulin at age 1 were associated with IgE sensitization at age 6.	Yes	(134)
1–13 years	Food and airborne PR-10 family of allergens.	Birch-atopic children developed a strong and persistent IgG response that preceded the IgE response to PR-10 allergens. Non-atopic children developed weak and transient IgG antibody response not involving IgE.	Yes	(135)
2 and 7 years	91 purified allergens, food and airborne.	The prevalence and magnitude of allergen-specific IgG at age 2, was higher in IgE-sensitized children than in non-sensitized children at ages 2 and 7	Yes	(136)
Children and adult	Airborne allergens.	High specific IgG antibodies were found in subjects with a positive specific IgE response. Low levels of specific IgG antibodies were found in subjects with no IgE response.	Yes	(137)
2–14 years	Airborne allergens: mite and cat.	IgE sensitization to mite allergens was correlated with exposure to mites and high specific IgG and IgG4.	Yes	(138)^*^
		High exposure to cat allergens without IgE sensitization was associated with high specific IgG and IgG4.	No	
Adults	Bee venom	Most beekeepers, who are frequently exposed to bee venom, develop high specific IgG4 antibody responses even in the absence of IgE sensitization.	No	(139)^*^

* *These manuscripts describe individuals with high allergen-specific IgG responses that are not associated with an IgE response*.

If antigen-specific human IgE is in fact generated from IgG precursors, antigen-specific IgG responses would precede and will be associated with antigen-specific IgE responses. In this context, it would be expected that IgE and IgG antibodies and plasma cells against the sensitizing allergen will be found in allergic individuals. This is in fact what has been found in several human studies. A longitudinal analysis of IgG and IgE antibodies to food (chicken ovalbumin, cow's milk β-lactoglobulin) and airborne allergens (mite and rye antigens) in children from 3 to 60 months of age detected specific IgG antibodies before the appearance of specific IgE antibodies (133). Another study followed a cohort of children at 6, 18 months, and 8 years of age. The authors found a positive correlation between IgE sensitization, clinical allergy, and high levels of specific IgG1 and IgG4 antibodies (23).

Based on cross-reactivities between airborne and food allergens, the Aalberse group hypothesized that an early IgG response against food allergens predisposes children to later IgE-sensitization to airborne allergens (19). The group found that at-risk children with high IgG against food allergens were more likely to develop IgE antibodies against airborne allergens. Recently the same group described detection of simultaneously occurring antigen-specific IgE and IgG against airborne allergens in allergic individuals (137).

Yet another study of allergic sensitization in children analyzed the correlation between IgA and IgG antibodies against wheat gliadin and cow's milk β-lactoglobulin at 1 year of age, and the development of IgE antibodies to food or inhaled allergens at age 6 (134). Increased IgA and IgG antibodies against gliadin or cow's milk β-lactoglobulin at age 1 were positively correlated with IgE sensitization in the child cohort at age 6. A recent longitudinal study of serum IgG and IgE antibody reactivity to the PR-10 family of allergens (which includes proteins in pollen and vegetable foods) was carried out in birch-allergic and non-atopic children from age 1–13 years (135). A weak and transient IgG antibody response, not involving IgE, was identified in non-atopic children. In contrast, birch-atopic children progressively developed a strong, and persistent IgG response that preceded an IgE atopic response to PR-10 allergens. The same group analyzed the correlation between IgG antibodies to a large panel of 91 purified allergens at age 2, and IgE sensitization at ages 2 and 7 (136). The authors found that both the prevalence and magnitude of allergen-specific IgG at age 2 was higher in IgE-sensitized children than in non-sensitized children at ages 2 and 7.

Thus, in general, high allergen-specific IgG responses during childhood precede or accompany IgE sensitization, and low IgG and IgA responses occur in non-allergic individuals (Table 2). There are some exceptions of high IgG/IgG4 responses without IgE sensitization in individuals exposed to high indoor levels of cat allergens (138), and in beekeepers repeatedly exposed to bee venom (139).

Importantly, the identification of Sγ remnants in human IgE switch regions has provided molecular validity for human IgG cells being the precursors of IgE plasma cells (107, 108, 113, 115). As discussed previously (see section Human IgE Cells), studies of the switch regions of human IgE cells isolated from peripheral blood (116, 117), or generated *in vitro* (119, 120), also identified Sγ remnants indicative of sequential switching. Consistent with the generation of human IgE cells by sequential switching, a high throughput DNA sequencing analysis of the IGH repertoire of allergic and healthy adults found that clonal lineages containing IgE members were predominantly related to IgG1 lineages (140). Sequential switching from IgG1 to isotypes other than IgE has also been described (40), but its biological significance is not yet known.

As we are proposing here that antigen-specific IgG cells are the precursors of pathogenic IgE in human allergy, we cannot ignore that IgG antibodies are also involved in protection from allergic reactions in patients that spontaneously outgrow allergies, and after successful immunotherapy (141). High levels of IgG antibodies, especially IgG4 antibodies, are found in sensitized patients that overcome allergies either spontaneously or after immunotherapy. IgG is believed to exert protection by sequestering the allergen and binding to inhibitory receptors, for example FcγRIIb in mast cells, as shown in mouse models (142). Other tolerance mechanisms are also involved in overcoming allergies, such the induction of Foxp3^+^ regulatory T cells, IL-10 secreting B regulatory cells and Tr1 cells. This topic is not discussed here, but excellent reviews can be found elsewhere (143–145).

In the section above we discussed the evidence that allergen-specific IgG responses precede IgE-sensitization in humans, and lack of IgE sensitization is mainly associated with weak allergen-specific IgG and IgA responses. These observations, together with the molecular marks of sequential switching from IgG to IgE, and the relatedness of IgE and IgG repertoires in allergic patients, are consistent with a model whereby human pathogenic IgE responses to allergens are in large part generated from allergen-specific IgG precursors.

## Concluding Remarks

We have accumulated considerable knowledge into the mechanics of the generation of IgE immune responses from studies in mice. These studies have demonstrated that mouse IgE cells follow a unique differentiation pathway characterized by an impaired germinal center phase, the predominance of the plasma cell phenotype, and a dependence on sequential switching to generate high affinity IgE. Furthermore, recent work has identified the subset of IgG1 memory cells that gives rise to high affinity IgE plasma cells.

While much less is known on the biology of human IgE cells, accumulating evidence suggests that the differentiation of human IgE cells is similar to that of mouse IgE cells. Several studies have shown that human IgE cells carry DNA footprints of sequential switching from IgG cells, and consistently, longitudinal studies in children found that allergen-specific IgG responses precede and are a risk factor for allergic sensitization. A few *in vivo* and *in vitro* studies have found that human IgE cells are predominantly plasma cells, and while circulating human IgE memory cells have been described, their function *in vivo* and their relevance for allergic disease is still unproven. Future studies utilizing the new cellular and genomic technologies, and improved humanized animal models, may shed new light on the origin, life span and unique characteristics of pathogenic IgE cells. These insights may help to design new therapies for allergic diseases.

## Author Contributions

All authors listed have made a substantial, direct and intellectual contribution to the work, and approved it for publication.

### Conflict of Interest Statement

The authors declare that the research was conducted in the absence of any commercial or financial relationships that could be construed as a potential conflict of interest.
